# Benefit-cost Trade-offs of Early Learning in Foraging Predatory Mites *Amblyseius Swirskii*

**DOI:** 10.1038/srep23571

**Published:** 2016-03-23

**Authors:** Inga C. Christiansen, Sandra Szin, Peter Schausberger

**Affiliations:** 1 Group of Arthropod Ecology and Behavior, Department of Crop Sciences, University of Natural Resources and Life Sciences Vienna, Peter Jordanstrasse 82, 1190 Vienna, Austria

## Abstract

Learning is changed behavior following experience, and ubiquitous in animals including plant-inhabiting predatory mites (Phytoseiidae). Learning has many benefits but also incurs costs, which are only poorly understood. Here, we addressed learning, especially its costs, in the generalist predatory mite *Amblyseius swirskii*, a biocontrol agent of several herbivores, which can also survive on pollen. The goals of our research were (1) to scrutinize if *A. swirskii* is able to learn during early life in foraging contexts and, if so, (2) to determine the costs of early learning. In the experiments, we used one difficult-to-grasp prey, i.e., thrips, and one easy-to-grasp prey, i.e., spider mites. Our experiments show that *A. swirskii* is able to learn during early life. Adult predators attacked prey experienced early in life (i.e., matching prey) more quickly than they attacked unknown (i.e., non-matching) prey. Furthermore, we observed both fitness benefits and operating (physiological) costs of early learning. Predators receiving the matching prey produced the most eggs, whereas predators receiving the non-matching prey produced the least. Thrips-experienced predators needed the longest for juvenile development. Our findings may be used to enhance *A. swirskii*’s efficacy in biological control, by priming young predators on a specific prey early in life.

Learning is defined as changed behavior following experience^1^,^2^. More precisely, learning comprises neuronal representations following information acquisition, and its retention and memory over time^3^. The ability to learn is ubiquitous and documented for many animals, both vertebrates and invertebrates^4^,^5^,^6^ including plant-inhabiting predatory mites of the family Phytoseiidae^7^,^8^,^9^,^10^,^11^. Learning allows animals to adjust their behaviors in changing environments and is generally assumed to have positive effects on fitness^5^. However, collecting, processing, and storing information, as well as recalling memory, and accumulating and connecting different information requires energy^12^,^13^. Thus, learning has not only benefits but also incurs costs. Accordingly, investing energy in learning is only selectively advantageous as long as the benefits outweigh the costs^13^,^14^. As compared to the benefits, the constitutive (maintenance of learning ability) and operating (information acquisition, storage, and retrieval) costs of learning are poorly understood^15^. Only recently, research on this topic, mainly using insects such as *Drosophila* spp., intensified.

Most pertinent studies on the benefit/cost trade-offs of learning were conducted with the fruit fly *Drosophila melanogaster* (Diptera, Drosophilidae). For example, Mery and Kawecki^16^,^17^,^18^ conducted several experiments with two outbred lines, a high-learning and a low-learning line originating from the same base population, and compared them concerning the benefit/cost trade-offs of learning. Learning by the flies had clear costs, e.g., under limited food availability, the high-learning line was better able to learn the aversive smell and taste of an oviposition substrate but showed poorer larval competitive abilities^16^. In another experiment^17^, the two outbred lines were exposed to different oviposition media in consecutive time cycles; flies from the conditioned high-learning lines laid fewer eggs than those from the low-learning lines. Mery and Kawecki^18^ also showed the costs of long-term memory after classical conditioning, associating an odor and an aversive mechanical shock. Conditioned flies died earlier in the absence of water and food than the control groups. An example of constitutive learning costs in butterflies (Lepidoptera) comes from *Pieris rapae*, where individuals with a better learning ability paid the costs of producing fewer eggs than those with a less well-developed learning ability^19^. Diet generalists like the grasshopper *Schistocerca americana* commonly need more time to choose their meal than diet specialists^12^. Accordingly, Bernays^12^ suggested that individuals having the ability to learn and process more information about different diets require more time for decision-making – thus a constitutive cost of learning – than individuals with a narrow food range and limited learning ability. The benefit of learning in these grasshoppers was an increase in growth rates^20^. In addition, learning and memory may interfere. Mensink and Raaijmakers^21^ distinguished between retroactive interference, when new information interferes with recalling old memories or proactive interference, which is when old memories interfere with the acquisition of new information. Memory interference is supposed to be costly and a factor selecting for short- or long-time diet specializations.

Here, we addressed learning, especially its costs, in the generalist predatory mite *Amblyseius swirskii* Athias-Henriot (Phytoseiidae). Phytoseiid mites are primarily living on plants, where they feed on other mites and small insects. They are widely used model animals in studies of behavior, ecology, and evolution^22^,^23^, including learning^7^,^8^,^9^,^10^,^11^. Recent studies suggested that these mites are, especially during the very early phases of life, after hatching and during the larval and early protonymphal stage, amenable to learning in foraging^8^,^9^ and social contexts^7^. Knowledge of the costs of learning in predatory mites is lacking.

The focal species of our study, *A. swirskii*, can feed on a broad range of prey, like spider mites, whiteflies, and thrips^23^,^24^,^25^,^26^, and is also able to utilize plant-derived substances such as pollen^27^,^28^. The origin of *A. swirskii* is the Mediterranean area^29^. In recent years, *A. swirskii* has become a commonly used natural enemy in augmentative biological control of thrips and whiteflies in greenhouse crops^23^,^25^. Due to its general feeding habits and use as biocontrol agent against diverse pest species, *A. swirskii* is an especially suitable species for learning studies. *Amblyseius swirskii* larvae are facultative feeders^30^, and, in general, the intensity of learning in foraging contexts is supposed to be stronger when the animals obtain feeding rewards^5^,^31^,^32^. In our study, we used two species of natural prey: the Western flower thrips *Frankliniella occidentalis* Pergande (Thripidae) and the two-spotted spider mite *Tetranychus urticae* Koch (Tetranychidae). Both herbivores belong to the most harmful crop pests worldwide^33^,^34^, feeding on a broad range of plants. *Frankliniella occidentalis* is primarily herbivorous but facultatively predaceous, has well developed anti-predator abilities^35^, and can also feed on eggs of spider mites^36^,^37^ and predatory mites^35^ including *A. swirskii* (personal observation). These habits make thrips a difficult-to-grasp, high-risk prey for *A. swirskii*. Foraging on thrips thus requires much higher energy investments by the predatory mites than foraging on spider mites, which are largely defenseless. Accordingly, *A. swirskii* can feed on all stages of the spider mites, but only on first instar thrips. The two prey species also differ in their body sizes-thrips are, depending on the life stage, 2 to 4 times larger than spider mites-and associated nutritional values for *A. swirskii*^23^,^38^,^39^. Since they can also be distinguished by their behavioral habits toward predatory mite attacks and the abilities of defending themselves, an attack by a predatory mite on thrips has presumably much higher costs than an attack on a spider mite.

The goals of our research were (1) to scrutinize if *A. swirskii* is able to learn during early life in foraging contexts and, if so, (2) to determine the benefits and costs of early learning for the predators. In the experiments, we used two prey species and types: one that is difficult to grasp and kill, i.e., thrips, and one that is easy to grasp and kill, i.e., spider mites. Thrips are a difficult prey because of their great mobility and counter-defense abilities. We hypothesized that the learning costs are evident in behavioral and/or life history traits^13^ and that learning thrips yields higher benefits but at the same time incurs higher costs than learning spider mites. Due to energetic and neural limitations, learning one type of prey comes at the cost of attacking, killing, and feeding on the other type of prey.

## Methods

### Experimental Animals, Population Origins, and Rearing

*Amblyseius swirskii* used in the experiments derived from two populations reared in the laboratory, both founded with specimens originating from Koppert (Koppert B.V., Veilingweg 14, 2651 AD Berkel en Rodenrijs, The Netherlands). The first experiment was performed in 2008, the second in 2014. The rearing unit for the population used in the first experiment consisted of a detached primary leaf of common bean, *Phaseolus vulgaris* L., placed upside down on a water-saturated foam cube, and dusted with maize pollen serving as food for the mites. The predators used in the second experiment were reared on artificial arenas consisting of plastic tiles (15 × 15 × 0.2 cm) resting on water-saturated foam in plastic boxes (20 × 20 × 6 cm) and surrounded by water-saturated tissue paper. They were fed at two to three day intervals with ‘Nutrimite’ (Biobest, Ilse Velden 18, 2260 Westerlo, Belgium), a diet exclusively consisting of cattail pollen *Typha angustifolia* L., by dusting pollen onto the arena. Additionally, small cotton tufts under cover slips were placed on the arena to provide shelter and oviposition sites for the predators.

Prey used in the experiments were first instar larvae of *F. occidentalis* and nymphs of *T. urticae. Frankliniella occidentalis* was reared on detached bean leaves of *P. vulgaris* (~11 × 13 cm) placed upside down on a 1% agar solution in a closed petri dish (14 cm Ø). For ventilation, a circular hole (1 cm Ø) was cut into the lid and covered with gauze. To obtain first instar larvae, adult thrips were randomly withdrawn from the stock population and transferred to a fresh bean leaf for 24 h for oviposition. After removing the adult thrips, the petri dish was stored in a climate chamber at 25 ± 1 °C, 65 ± 5% relative humidity (RH), and 16:8 h L:D photoperiod for 3.5 d. At that time, most larvae had hatched and the petri dish was kept in a fridge at 8 °C and darkness to stop any further development of thrips larvae. Only first instar larvae were used as prey in the experiments.

*Tetranychus urticae* was reared on whole bean plants *P. vulgaris.* Plants were grown at room temperature 23 ± 2 °C and 16:8 h L:D photoperiod. For the experiments, only proto- and deutonymphs were used as prey. The spider mites were manually brushed from infested leaves using a paint brush or using a mite brushing machine (BioQuip^®^, 2321 Gladwick Street, Rancho Dominguez, CA 90220, USA) onto glass plates and then singly picked up and placed into acrylic cages using a fine red marten’s hair brush.

All rearing and experimental units were kept in a climate chamber at 25 ± 1 °C, 65 ± 5% RH and 16:8 h L:D photoperiod.

### Experimental Procedures

To obtain even-aged eggs of *A. swirskii,* giving rise to experimental individuals, gravid females were randomly withdrawn from the stock populations and placed on a fresh detached bean leaf arena with pollen. Eggs were collected once a day with a fine brush, placed singly into cylindrical cages of 15 mm diameter and 3 mm height, laser-cut into rectangular acrylic plates, closed with fine gauze at the bottom and a microscope slide on the upper side^40^. Cages were stored upside down on a grid in an open plastic box, the bottom of which was filled with water to warrant elevated humidity inside the cages^39^.

### Early Learning Ability (Experiment 1)

The first experiment aimed at examining whether *A. swirskii* can learn a given prey species early in life. The cages, each containing a single *A. swirskii* egg, were loaded with prey to be present during the larval and early protonymphal stage. The cages were randomly assigned to be loaded with either five nymphs of *T. urticae*, or three first instar larvae of *F. occidentalis,* or a combination of three *T. urticae* nymphs and three *F. occidentalis* larvae (Table 1). After hatching, the predatory mite were allowed to contact and feed on prey in the larval and early protonymphal stage. Shortly after the predatory mites had developed to protonymphs, they were transferred to new cages and provided with either five spider mite nymphs or three first instar thrips larvae, replenished once per day, until reaching adulthood (Table 1). Adult female predators were placed together with a male, randomly taken from the rearing, into the experimental cage and provided with either ten spider mite nymphs or ten thrips larvae, resulting in predators receiving prey matching or non-matching the one experienced early in life, or being intermediate (i.e., having experienced both the matching and an alternative prey) (Table 1). The intermediate treatment was included to determine whether simultaneous presence of both prey types interferes with learning one prey type by *A. swirskii.* Subsequently, the attack latency was assessed by taking the time elapsed until the first successful attack on prey by the female predatory mite. Attacks on thrips were exclusively launched by the females but never the males (due to size constraints). After 24 h, the male was removed from the cage and discarded. Attacks were scored successful when the predators had grasped and started to suck on prey. Time until the first attack occurred was recorded in minutes. For the first 15 min, mites were observed constantly without interruption. Subsequently, spot observations in 2 min intervals were carried out until the first successful attack occurred. Each of the six treatments was replicated 12 times.

### Benefits and Costs of Early Learning (Experiment 2)

To assess the costs of early learning a given prey, the second experiment consisted of three phases: experience, consolidation, and behavioral assay. To start the experience phase, the cages, each containing a single *A. swirskii* egg, were loaded with prey and/or pollen to be present during the sensitive larval and early protonymphal stage. The cages were randomly assigned to be loaded with either two nymphs of *T. urticae*, or two first instar larvae of *F. occidentalis,* or just pollen or a combination of two *T. urticae* nymphs and pollen, or two *F. occidentalis* larvae and pollen. Cages were checked twice daily at 8 h intervals. Thrips occasionally killed predator eggs but never larvae or protonymphs. After hatching, the predatory mites were allowed to contact and feed on prey and/or pollen in the larval and early protonymphal stage. The experience phase lasted until the predator reached the protonymphal stage and on average one day. Dead prey individuals found in the cage during the experience phase were taken as evidence of successful attacks by the predatory mites. For consolidation of their early prey experiences, the protonymphs were then transferred to a new cage, using a moistened red marten’s hair brush, and only fed with pollen until reaching adulthood. Depending on the developmental time, the consolidation phase lasted 4 to 6 d. After the mites reached adulthood, their sex was determined and males were discarded. Adult females were transferred to a new cage together with a male, randomly taken from the rearing, for mating and left without food but access to free water. To provide free water, each cage was equipped with a strip of filter paper tightly attached to the gauze on the bottom of the cage on one end and reaching into tap water with the other end. After 24 h, the male was removed and the behavioral assay carried out. To this end, each female was provided with two prey items, either two *T. urticae* nymphs or two *F. occidentalis* larvae. After loading, the time until the first successful attack (encounter and kill) of the predator was taken by checking the cages every 10 min for the first two h and every 30 min in the third h. After 24 h, the number of killed prey was noted and prey, alive or dead, was removed from the cage. Females were then left without any food but access to free water until natural death of the female. To monitor female survival and count and remove laid eggs, the cages were checked in 24 h intervals.

Two types of prey offered to adult females that during early life had experienced either *T. urticae*, or *F. occidentalis*, or pollen, or *T. urticae* and pollen, or *F. occidentalis* and pollen, resulted in ten treatments in total. Conclusively, regardless of the prey species offered in the behavioral assay, there was one group of predators receiving the familiar prey (called matching prey) when adult, one group receiving an unfamiliar prey (called non-matching prey), and one control group (called neutral), which did not experience any prey during early life, because only fed on pollen, and offered one of the two prey species when adult. Each of the ten treatments was replicated 16 to 21 times. To minimize observer bias, blind methods were used when the behavioral data were recorded.

### Statistical Analyses

Statistical analysis was carried out using PASW Statistics 18 (SPSS Inc.). In the first experiment, we used a generalized linear model (GLM) to analyze the influence of prey matching (non-matching, matching, or intermediate prey) and prey species offered (spider mites or thrips) on the attack latency (normal distribution, identity link) of adult predator females. Pairwise differences were assessed by least significant difference (LSD) tests. In the second experiment, treatments with and without additional pollen in the cages with prey during the early learning phase were analyzed separately, with the control group (only pollen) used for both treatment categories. Separate GLMs were used to analyze the influence of (1) larval experience with spider mites, thrips, or pollen and predator sex on total developmental time, and developmental time during the larval/protonymphal learning phase and during consolidation, i.e., from late protonymphs to adult (normal distribution, identity link), (2) larval experience with spider mites or thrips and prey attack in the learning phase (yes/no) on total developmental time (normal distribution, identity link), (3) prey matching (matching, non-matching, and neutral), prey attack in the learning phase (yes/no) and prey offered to adult predators (spider mites or thrips) on the attack latency (normal distribution, identity link), and (4) prey matching (matching, non-matching, and neutral) and prey offered to adult predators (spider mites or thrips) on egg production (Gamma distribution, log link) and longevity (Poisson distribution, log linear).

## Results

### Early Learning Ability (Experiment 1)

**Figure 1:**
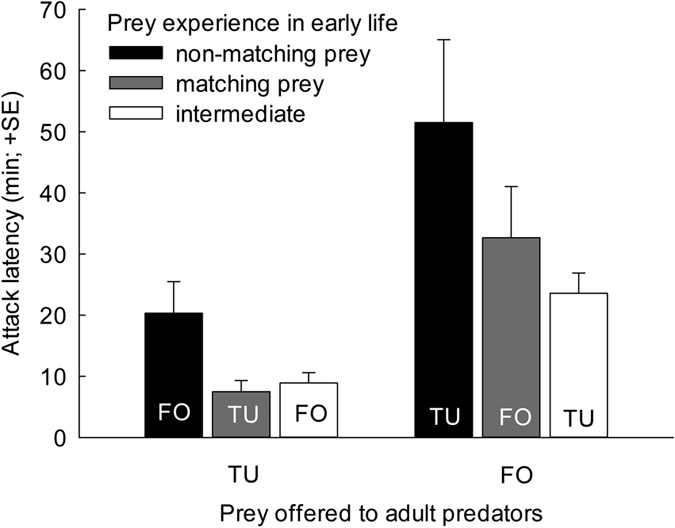
Attack latency of adult *A. swirskii* females on either spider mite nymphs or first instar thrips. The predators experienced early in life, during the larval and early protonymphal stage, either spider mites *T. urticae* (TU), thrips *F. occidentalis* (FO), or a combination of spider mites and thrips (intermediate). Before reaching adulthood, they were fed with either spider mites or thrips (letters within bars) and, after reaching adulthood, they received either the prey species matching or non-matching the prey experienced during early life.

Prey experience early in life, in the larval and early protonymphal stage, affected the attack latency of *A. swirskii* as adult females (Fig. 1). No matter whether the predators had experienced spider mites or thrips early in life, adult female predators attacked the matching prey, i.e., the prey they had experienced early in life, more quickly than a non-matching, unfamiliar prey (GLM; Wald χ_2_^2^ = 9.571, *P* = 0.008). Pairwise comparisons revealed that the non-matching prey treatment differed from the matching and intermediate treatment (LSD; *P* < 0.05), whereas the intermediate and matching prey treatments were similar (*P* = 0.57). The predatory mite females attacked the spider mites across treatments more quickly than they attacked thrips (GLM; Wald χ_1_^2^ = 18.495, *P* < 0.001). The interaction of prey species offered to adult predators and prey matching was non-significant (GLM; Wald χ_2_^2^ = 1.535, *P* = 0.46).

### Benefits and Costs of Early Learning (Experiment 2)

In the early learning phase, the additional presence of pollen in the cages with prey strongly affected the likelihood that prey was attacked by the adult female predators in the attack latency test (GLM; Wald χ_1_^2^ = 10.043, *P* = 0.002). Predators that were additionally provided with pollen during the learning phase, attacked prey less likely (33.33% ± 4.6) than those exclusively provided with prey (54.87% ± 4.7). Thus, all subsequent analyses were conducted separately for treatments with prey and additional pollen and treatments with prey without pollen. In both treatment categories, the pure pollen group served as control.

**Figure 2:**
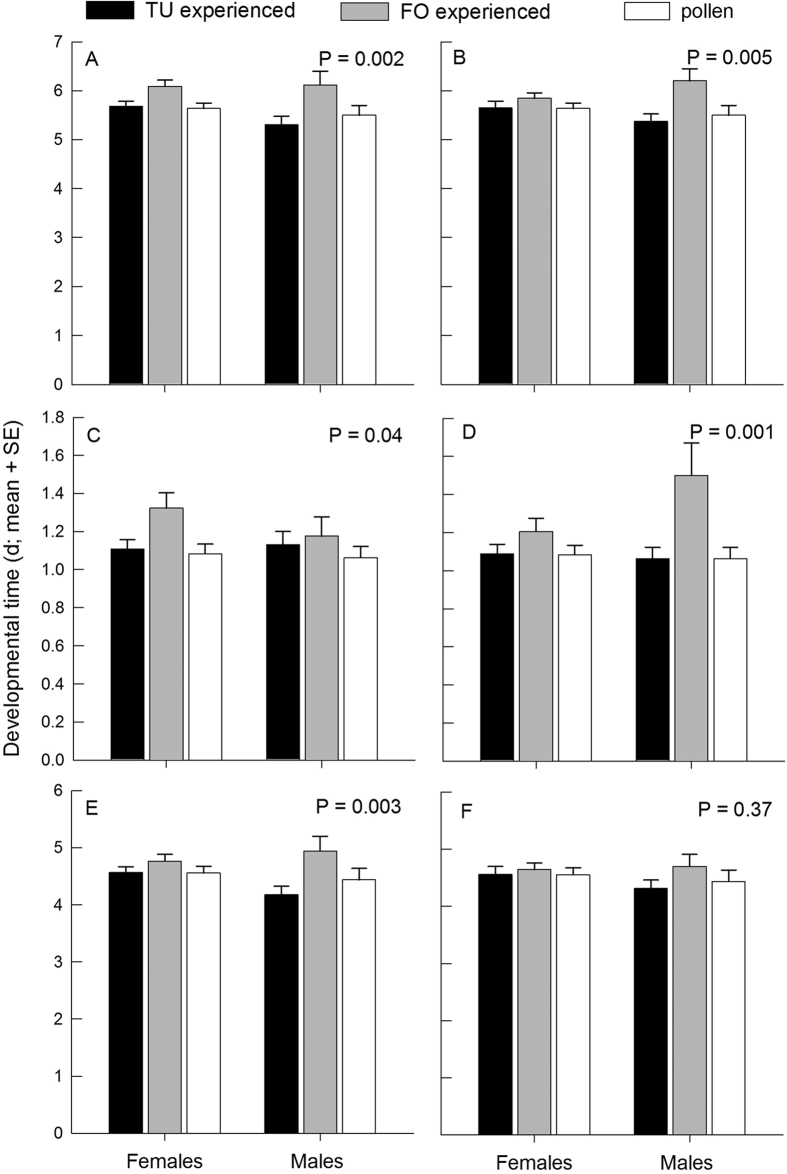
Developmental times of female and male *A. swirskii*, in total from larvae to adult (**A,B**), during the experience phase in the larval and early protonymphal stage (**C,D**), and during the consolidation phase (**E,F**). The predators experienced early in life, during the larval and early protonymphal stage, either spider mites (TU) or thrips (FO), either without (**A,C,E**) or with (**B,D,F**) additional pollen, or no prey (only pollen) and were then fed on pollen until reaching adulthood (consolidation phase). P values within graphs refer to the difference in developmental time (GLM) between thrips-experienced predators and spider mite-experienced predators and pollen fed predators.

Early prey experience affected the developmental time of *A. swirskii* (Fig. 2). Predators experiencing thrips early in life needed longer for total development, from larva to adult, than those experiencing spider mites and those without any prey experience (GLMs; without additional pollen: Wald χ_2_^2^ = 12.009, *P* = 0.002; with additional pollen: Wald χ_2_^2^ = 10.478, *P* = 0.005). Neither sex (without additional pollen: Wald χ_1_^2^ = 1.263, *P* = 0.26; with additional pollen: Wald χ_1_^2^ = 0.020, *P* = 0.89) nor the interaction of early prey experience and sex (without additional pollen: Wald χ_2_^2^ = 1.377, *P* = 0.50; with additional pollen: Wald χ_2_^2^ = 3.702, *P* = 0.16) had an effect on the total developmental time (Fig. 2A,B). Thrips experience prolonging development of the predators was true for both treatment categories, with and without additional pollen in the larval/protonymphal learning phase (without additional pollen: Wald χ_2_^2^ = 6.326, *P* = 0.042; with additional pollen: Wald χ_2_^2^ = 16.866, *P* < 0.001) (Fig. 2C,D) but was only true for treatments without additional pollen in the consolidation phase (without additional pollen: Wald χ_2_^2^ = 11.526, *P* = 0.003; with additional pollen: Wald χ_2_^2^ = 1.999, *P* = 0.37) (Fig. 2E,F). Neither sex nor the interaction of early prey experience and sex (without or with additional pollen: *P* > 0.05 for all analyses) had an effect on the developmental time during the learning and consolidation phase (Fig. 2C–F). Prey attack during the learning phase (yes/no) did not affect the total developmental time (without additional pollen: d, mean ± SE; attack yes = 5.804 ± 0.127; attack no = 5.825 ± 0.139; Wald χ_1_^2^ = 0.013, *P* = 0.91; with additional pollen: attack yes = 5.679 ± 0.156; attack no = 5.783 ± 0.097; Wald χ_1_^2^ = 0.321, *P* = 0.57), independent of prey type, as indicated by the non-significant interaction with early prey experience (without additional pollen: Wald χ_1_^2^ = 0.001, *P* = 0.97; with additional pollen: Wald χ_1_^2^ = 0.031, *P* = 0.86).

**Figure 3:**
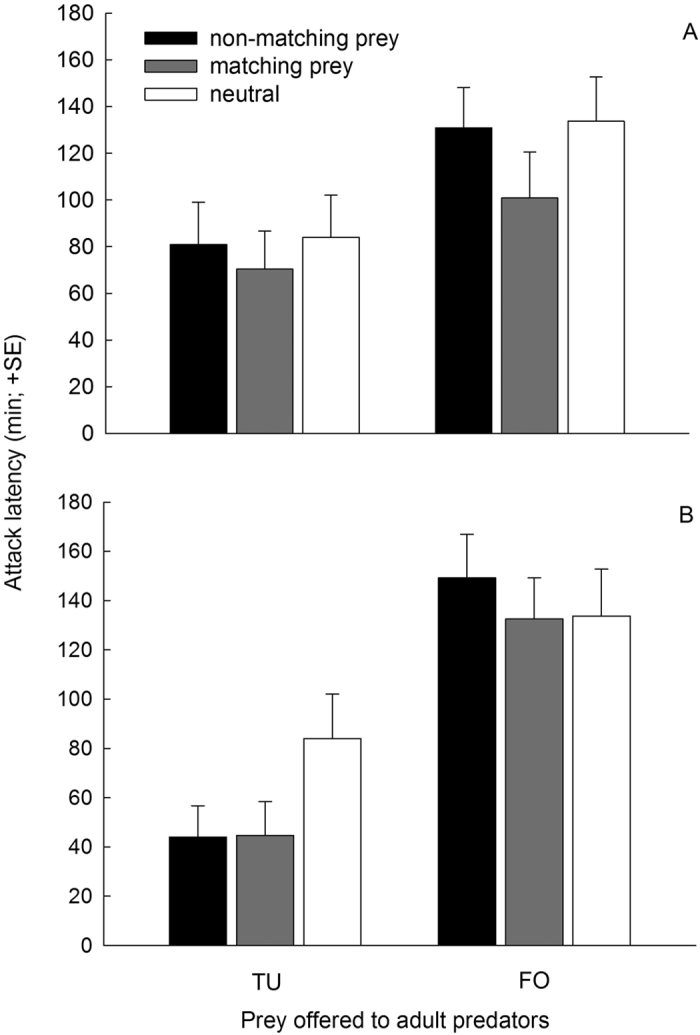
Attack latency of gravid *A. swirskii* females on either spider mite nymphs or first instar thrips. The predators experienced early in life, during the larval and early protonymphal stage, either spider mites (TU) or thrips (FO), either without (**A**) or with (**B**) additional pollen, or only pollen (neutral) and were then fed on pollen until reaching adulthood. Prey species (TU or FO) offered to adult females either matched or did not match the prey species experienced early in life.

In both treatment categories, without (Fig. 3A) and with (Fig. 3B) additional pollen, *A. swirskii* attacked the spider mites more quickly than they attacked thrips. However, *A. swirskii* that had successfully attacked prey in early life attacked thrips but not spider mites more quickly than those that had only contacted prey in early life. In the treatment category without additional pollen, adult predators attacked prey matching their early prey experience more quickly than did naïve predators (neutral) and predators offered non-matching prey, which had similar attack latencies (Fig. 3A; Table 2). In the treatment category with additional pollen during the early learning phase, the attack latencies were not influenced by prey matching (Fig. 3B; Table 2).

**Figure 4:**
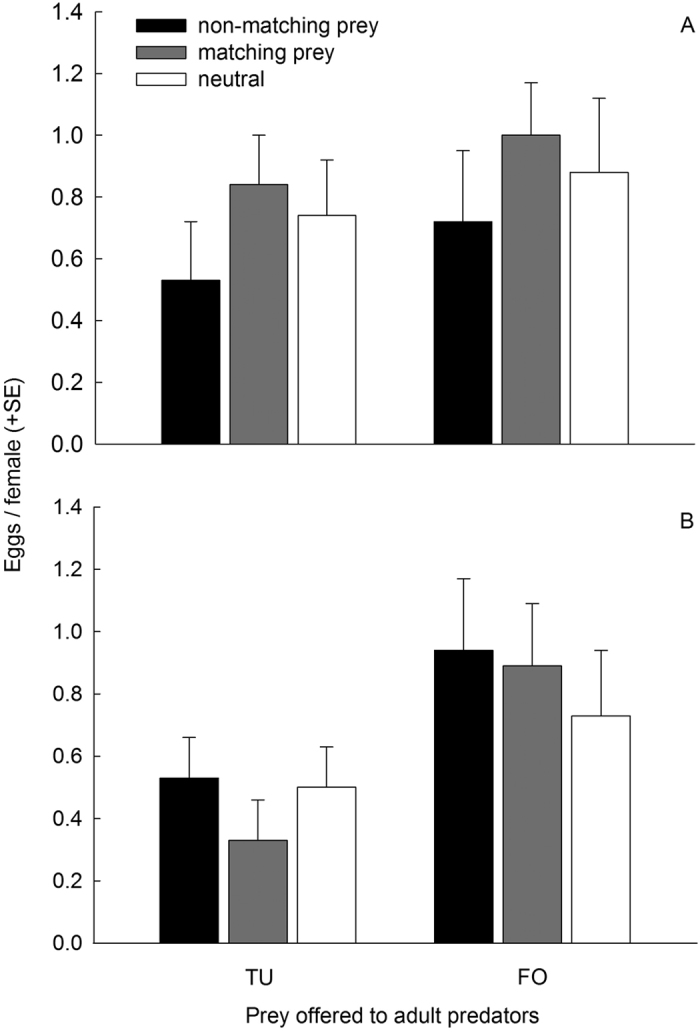
Number of eggs laid per *A. swirskii* female, after the attack latency test, until natural death. The predators experienced early in life, during the larval and early protonymphal stage, either spider mites (TU) or thrips (FO), either without (**A**) or with (**B**) additional pollen, or only pollen (neutral) and were then fed on pollen until reaching adulthood. In the attack latency test the predators were offered either spider mite nymphs (TU) or first instar thrips (FO), which either matched or did not match the prey species experienced early in life.

In both treatment categories, without (Fig. 4A) and with (Fig. 4B) additional pollen, *A. swirskii* offered thrips produced more eggs than those offered spider mites (GLMs; without additional pollen: Wald χ_1_^2^ = 5.257, *P* = 0.022; with additional pollen: Wald χ_1_^2^ = 8.924, *P* = 0.003). In the treatment category without additional pollen, predator females receiving matching prey laid the most eggs, whereas those receiving non-matching prey laid the fewest eggs (Wald χ_2_^2^ = 6.037, *P* = 0.049). In contrast, egg production was not influenced by prey matching in the treatment category with additional pollen (Wald χ_2_^2^ = 0.771, *P* = 0.68). The interaction of prey offered to adult predators and prey matching did not have an effect (without additional pollen: Wald χ_2_^2^ = 0.141, *P* = 0.93; with additional pollen: Wald χ_2_^2^ = 1.082, *P* = 0.58).

**Figure 5:**
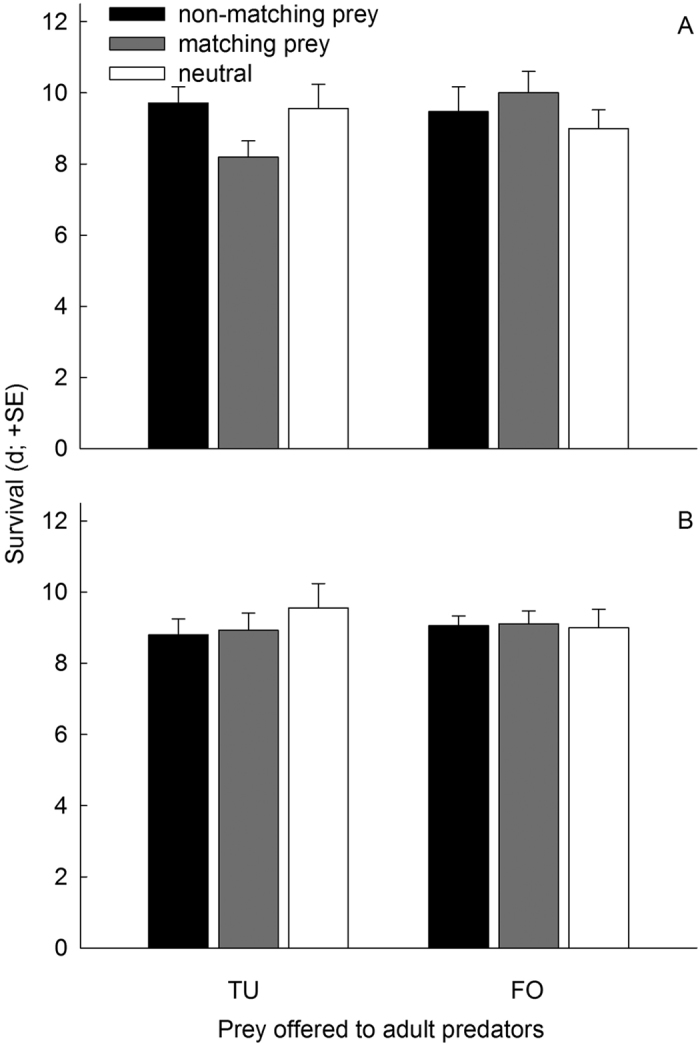
Longevity of *A. swirskii* females held without prey following the attack latency test. The predators experienced early in life, during the larval and early protonymphal stage, either spider mites (TU) or thrips (FO), either without (**A**) or with (**B**) additional pollen, or only pollen (neutral) and were then fed on pollen until reaching adulthood. In the attack latency test, the predators were offered either spider mite nymphs (TU) or first instar thrips (FO), which either matched or did not match the prey species experienced early in life, and were then held without food, but with access to free water until natural death.

Longevity of adult predators, without food following the attack latency tests, ranged between 8 to 10 d across treatments. In neither treatment category, without (Fig. 5A) and with (Fig. 5B) additional pollen, longevity was influenced by prey offered to adult predators (GLMs; without additional pollen: Wald χ_1_^2^ = 0.607, *P* = 0.44; with additional pollen: Wald χ_1_^2^ = 0.009, *P* = 0.93) or prey matching (without additional pollen: Wald χ_2_^2^ = 1.005, *P* = 0.61; with additional pollen: Wald χ_2_^2^ = 0.524, *P* = 0.77).

## Discussion

Our study documents the ability of the predatory mite *A. swirskii* to modify foraging by experience and provides evidence for physiological, behavioral, and cognitive benefits and costs. The first experiment shows that *A. swirskii* can learn in a sensitive phase, as larvae and early protonymphs, like other predatory mites do, e.g., *Neoseiulus californicus* in foraging contexts^9^ and *Phytoseiulus persimilis* in social^41^ and cannibalism^8^ contexts. Memory was retained through two molting events and was successfully recalled by adult predatory mites. *Amblyseius swirskii* that had learned a specific prey early in life – in this case either spider mites or thrips – attacked later in life, as adult females, the prey matching their early experience more quickly than they attacked non-matching prey. The second experiment corroborates the findings that early prey experience shortens the attack latencies on matching prey and additionally indicates both fitness-relevant benefits and operating (physiological) costs of early learning. Predators receiving the matching prey produced the most eggs, whereas predators receiving the non-matching prey produced the fewest. Thrips-experienced predators needed longer for juvenile development, both during the learning and consolidation phase, than predators experiencing the easy-to-grasp prey spider mites and neutral, i.e., pollen-exposed, predators. Neither experiment provided evidence for cognitive costs.

Learning may affect predatory mites in all major life activities, i.e., in foraging using herbivore-induced plant volatiles, HIPVs^42^,^43^,^44^,^45^, or direct prey cues^9^, mating^46^, anti-predator behavior^10^, and social interactions^11^,^47^. Based on the rationale that across animals, early in life the neural system is much more plastic than later on, it is mostly the very early life stages of predatory mites, larvae and protonymphs, which have been tested for learning in foraging and social contexts. For example, *N. californicus* may imprint on prey contacted in early life, which represents a non-associative learning mechanism^9^. It can feed on spider mites and thrips, but similar to our results for *A. swirskii*, when the predators had experienced thrips early in life, they attacked thrips more quickly and fed more on thrips later in life^9^. *Phytoseiulus persimilis,* a specialist predator of spider mites, can, for example, discriminate familiar and unfamiliar individuals based on contact early in life, in cannibalism, foraging, and group-living contexts^11^,^40^,^41^,^47^. Regarding prey usage and detection, experience may improve the performance of *P. persimilis* in cannibalism^8^ and response to HIPVs^43^. Across pertinent studies, an apparent common effect of early prey experience is improvement of direct prey recognition and consequently shorter attack latencies.

Our study provides evidence of the fitness benefits of learning in *A. swirskii*. Predators that had learned a specific prey during early life and then received the same prey as adult females laid more eggs than predators receiving non-matching prey as adults, counterbalancing and likely outweighing the costs of longer juvenile development. This finding supports the assumption that early learning is especially advantageous when the environments of juvenile and adult animals are matching^13^,^14^,^48^,^49^. Proximately, experienced predators wasted less energy in finding, attacking, and handling prey and thus could allocate more energy to egg production. Egg laying rates are an especially appropriate measure for the benefits and costs of learning. For example, in *D. melanogaster,* the operating costs of repeated learning were most evident in a reduced egg laying rate^17^.

Thrips-experienced predators needed longer for juvenile development than spider-mite-experienced and prey-naïve (neutral) predators. They were not able to catch-up or compensatory growth as shown for the predatory mite *P. persimilis*^50^, following the stressful thrips learning phase in the larval and early protonymphal stage. The presence and interaction with the difficult-to-grasp prey thrips and internal consolidation of this experience apparently required more energy^12^,^13^,^51^ than other, less stressful experiences and subsequent consolidation during development. This energy was traded-off against investment in developmental progress, and consequently prolonged the developmental times of the predators. During the experience phase, thrips-exposed predators with and without additional pollen needed longer for development. In contrast, during the consolidation phase, only predators having experienced thrips without pollen but not those with additional pollen needed longer for development. This finding supports our explanation of a prolonged developmental time reflecting the operating costs of learning. While one might argue that during the experience phase the response to the mere presence of the difficult-to-grasp thrips prolonged development, during the consolidation phase all predators, independent of their prey experience early in life, received the same food, i.e., pollen. However, only those having experienced thrips before invested energy to strengthen existing or to establish new neural connections and thus required more time to reach adulthood. Regarding longevity, we did not detect any physiological costs of learning. Constitutive (genetic) costs of learning ability were observed in *D. melanogaster.* Flies selected for an improved learning ability lived shorter than those with a reduced learning ability, indicating evolutionary trade-offs between cognitive and life history traits^15^. Within the high-learning line, conditioned flies were less productive in laying eggs than unconditioned flies, providing an example for operating costs of learning^17^. An example for operating costs of learning in vertebrates comes from mice, where learning reduced immunity^52^.

Learning requires energy to strengthen existing or to establish new neural connections or build and restore neurons and generate signals^51^. Thus, due to limitation of the neural system, learning improving the cognitive performance in one behavioral task may come at the cost of a reduced performance in other tasks^13^. Cognitive constraints, for example, were observed in honeybees paying the costs of learning by suffering from retention deficits when energetically stressed^53^. In our study, we did not detect any cognitive costs in *A. swirskii*. In the second experiment, cognitive costs could have been evident if learning, which occupies a given portion of the neural system, would have led to better performance with the matching prey but worse performance with the non-matching prey than prey-naïve (neutral) predators. However, predators receiving the non-matching prey did not differ in attack latencies from naïve mites, i.e., those without any prey experience before tests. Using a modeling approach, Clark and Dukas^54^ evaluated the complex interplay of the cognitive costs and benefits of information processing in predators and predicted costs, due to limited attention, when available information exceeds the threshold of the processing capacity of the brain. Limited attention is also one plausible explanation for the absence of learning by young *A. swirskii* kept in cages with prey and additional pollen. Their attack latencies on matching and non-matching prey did not differ. An information or signal overload^51^ or signal overshadowing^55^,^56^ might have constrained the prey learning effect under these conditions. Alternative or additional explanations are that the predators had no need to learn prey because the easy-to-get pollen was abundantly available, they were less motivated to learn prey because being satiated by feeding on pollen, or that the contact with prey, i.e., the encounter frequency, was too low to induce learning. Pinpointing the causes of additional pollen presence compromising learning a given prey requires further scrutiny.

The here presented results show that early learning by *A. swirskii* may prime the predators on a specific prey experienced early in life, as larvae and early protonymphs. In natural settings, this learning ability is useful in matching environments experienced during early life and after reaching adulthood, i.e., where the presence of a specific prey does not change during ontogeny of the predator, because the mites are then better able to attack and handle the learned prey. In non-matching environments, after reaching adulthood, the learning costs should not be too high because in our experiments, the experienced predators did not perform worse with the non-matching prey than did naïve predators. Before reaching adulthood, learning incurred costs, which was evident in the prolonged developmental time. In matching environments, learning is adaptive because it translates into the fitness benefit of a higher reproduction rate, which should cascade up the organizational levels and enhance population growth. In agricultural settings, priming predators on a specific prey, especially a difficult-to-grasp prey like thrips, can be exploited to improve the performance of these predators in biological control^57^,^58^. Predatory mites commercially used in biological control of thrips, including *A. swirskii,* are commonly mass-reared on factitious food or other than target prey^59^ and thus do not experience thrips before release, possibly compromising their efficacy against this pest. Finding a way to prime the predators on their target pest is an exciting future challenge for commercial production of predatory mites and their use in biological control.

## Additional Information

**How to cite this article**: Christiansen, I. C. *et al*. Benefit-cost Trade-offs of Early Learning in Foraging Predatory Mites *Amblyseius Swirskii. Sci. Rep.* **6**, 23571; doi: 10.1038/srep23571 (2016).

## Figures and Tables

**Table 1:** Prey treatments used in the first experiment to determine early learning ability in the predatory mite *A. swirskii.*

Prey matching (early learning phase and test with adult females)	Prey during early learning phase (larvae and early protonymphs)	Prey during consolidation phase (late protonymphs and deutonymphs)	Prey offered to adult females during testing
non-matching	thrips	thrips	spider mites
matching	spider mites	thrips	spider mites
intermediate	spider mites + thrips	thrips	spider mites
non-matching	spider mites	spider mites	thrips
matching	thrips	spider mites	thrips
intermediate	spider mites + thrips	spider mites	thrips

**Table 2:** Results of generalized linear models (GLM) on the influence of prey matching (matching, non-matching or neutral), prey attack in the learning phase (yes/no) and prey species offered to adult predators (spider mites or thrips) on the attack latency in treatment categories without or with additional pollen in the early learning phase.

Source of variation	Wald χ^2^	df	*P*
Without pollen			
Matching prey	4.796	1	0.029
Prey attack	<0.001	1	0.992
Prey species	6.858	1	0.009
Prey species*matching prey	0.382	1	0.537
Prey species*prey attack	4.142	1	0.042
Matching prey*prey attack	0.437	1	0.508
With pollen			
Matching prey	1.335	1	0.248
Prey attack	0.062	1	0.803
Prey species	27.938	1	<0.001
Prey species*matching prey	0.571	1	0.45
Prey species*prey attack	3.674	1	0.055
Matching prey*prey attack	0.003	1	0.953
