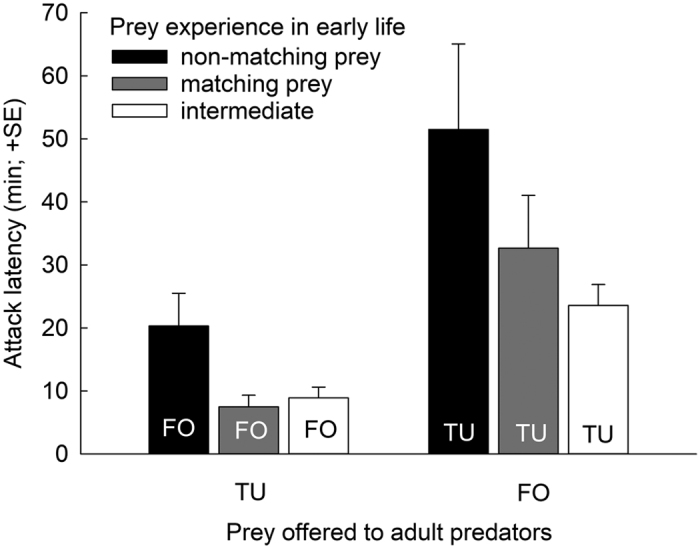# Corrigendum: Benefit-cost Trade-offs of Early Learning in Foraging Predatory Mites *Amblyseius Swirskii*

**DOI:** 10.1038/srep46948

**Published:** 2018-03-01

**Authors:** Inga C. Christiansen, Sandra Szin, Peter Schausberger

Scientific Reports 6: Article number: 2357110.1038/srep23571; published online: 03 23 2016; updated: 03 01 2018

This Article contains an error in Figure 1 where the labelling within the ‘matching prey’ bars were inverted. The correct Figure 1 appears below.

## Figures and Tables

**Figure 1:**